# Dynamic Regulation of Sarcomeric Actin Filaments in Striated Muscle

**DOI:** 10.1002/cm.20476

**Published:** 2010-08-09

**Authors:** Shoichiro Ono

**Affiliations:** Department of Pathology and Department of Cell Biology, Emory UniversityAtlanta, Georgia

**Keywords:** myofibrils, sarcomeres, actin turnover, congenital myopathy, stabilization, depolymerization, capping

## Abstract

In striated muscle, the actin cytoskeleton is differentiated into myofibrils. Actin and myosin filaments are organized in sarcomeres and specialized for producing contractile forces. Regular arrangement of actin filaments with uniform length and polarity is critical for the contractile function. However, the mechanisms of assembly and maintenance of sarcomeric actin filaments in striated muscle are not completely understood. Live imaging of actin in striated muscle has revealed that actin subunits within sarcomeric actin filaments are dynamically exchanged without altering overall sarcomeric structures. A number of regulators for actin dynamics have been identified, and malfunction of these regulators often result in disorganization of myofibril structures or muscle diseases. Therefore, proper regulation of actin dynamics in striated muscle is critical for assembly and maintenance of functional myofibrils. Recent studies have suggested that both enhancers of actin dynamics and stabilizers of actin filaments are important for sarcomeric actin organization. Further investigation of the regulatory mechanism of actin dynamics in striated muscle should be a key to understanding how myofibrils develop and operate. © 2010 Wiley-Liss, Inc.

## Introduction

Actin is one of the major cytoskeletal proteins in eukaryotic cells and plays essential roles in a number of cellular processes including cell migration, cytokinesis, vesicle transport, and contractile force generation [Pollard and Cooper, 2009]. Recent advancements in live cell imaging techniques have revealed dynamic aspects of the actin cytoskeleton in a number of cell biological events. Myofibrils in striated muscle are not exceptions to the dynamic actin cytoskeleton. Striated myofibril is one of the most differentiated forms of the actin cytoskeleton, in which actin, myosin, and other regulatory components are organized into sarcomeres and produce contractile forces in a calcium-regulated manner [Squire, 1997; Clark et al., 2002]. Skeletal and cardiac muscles in vertebrates are representative striated muscle, and a number of invertebrates also have transversely or obliquely striated muscle [Hooper et al., 2008]. It is actually surprising to realize that a number of sarcomeric proteins within actively contracting myofibrils undergo dynamic turnover without compromising overall organization [Littlefield and Fowler, 2008; Sanger and Sanger, 2008]. In particular, sarcomeric actin filaments are aligned with similar lengths and yet exhibit dynamic exchange of actin subunits within the filaments. Dynamics of sarcomeric actin filaments have been described in a number of reports, and some regulatory mechanisms have been revealed. Recent studies have suggested a link between actin dynamics and muscle diseases. This review summarizes current understanding of the regulation of actin filament dynamics in striated muscle and its biological significance.

## Arrangement of Actin Filaments in Sarcomeres

**Fig. 1:**
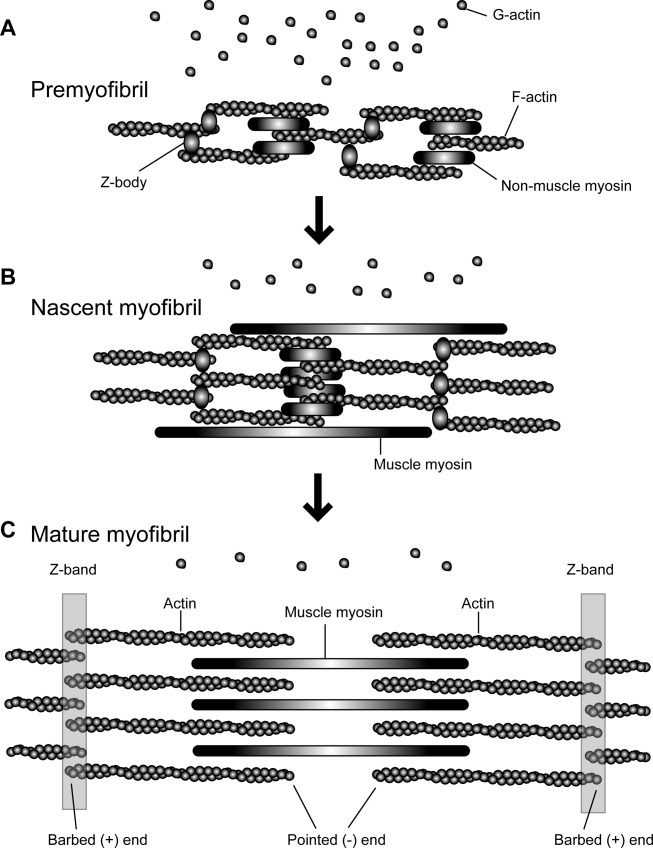
Actin assembly and organization during myofibrillogenesis The assembly process of myofibrils is based on the premyofibril model proposed by Sanger et al. [2006, 2010]. In premyofibrils (**A**), actin filaments are nonstriated and associated with the Z-bodies containing α-actinin and nonmuscle myosin filaments. At this stage, high concentrations of G-actin are present. In nascent myofibrils (**B**), alignment of the Z-bodies and nonmuscle myosin becomes more ordered, while actin filaments are still nonstriated. Muscle myosin is also assembled in a nonstriated manner. The ratio of G-actin to F-actin likely decreases during this transition, although a precise correlation of the assembly stages and G/F-actin ratios has not been reported. In mature myofibrils (**C**), actin filaments, muscle myosin filaments, and Z-bands are organized in sarcomeres. G-actin concentrations drop to ∼1% of total actin, whereas 99% of actin is present in myofibrils as F-actin. Note that size and compositions of other myofibril proteins vary in different muscle types and organisms.

Sarcomeres are composed of regularly aligned thin and thick filaments for efficient production of contractile forces (Fig. 1C). Myosin-based thick filaments have myosin heads in bipolar orientations and registered in the middle of sarcomeres. Actin-based thin filaments are oriented in opposite directions at each end of a sarcomeric unit (Fig. 1C), which is essential for production of contractile forces by unidirectional movement of the myosin motors. The barbed ends of actin filaments are anchored to the Z-bands that separate each sarcomeric unit (Fig. 1C). α-actinin crosslinks actin filaments near their barbed ends in the Z-bands and stabilizes polarized registration of the thin filaments. On the other hand, the pointed ends of actin filaments are not bound to a particular structure (Fig. 1C). Nonetheless, uniform lengths of thin filaments are maintained in striated muscle. The mechanism of the regulation of thin filament length is still a major unsolved problem and is discussed in other review articles [Littlefield and Fowler, 1998, 2008].

Cardiac and skeletal muscles in vertebrates are transversely striated (cross-striated) muscle in which thin and thick filaments are laterally aligned perpendicularly to the Z-bands (or Z-discs). Thin filaments in skeletal muscle are relatively constant in length, while those in cardiac muscle are present in variable lengths [Robinson and Winegrad, 1977, 1979]. Sarcomeres are delineated by the Z-bands that are linked to the plasma membrane through attachment structures, designated as costameres [Ervasti, 2003]. Among invertebrates, arthropods have similarly organized cross-striated muscle. Therefore, the indirect flight muscle of the fruit fly *Drosophila melanogaster* has been used as a model to study assembly and function of cross-striated myofibrils [Fyrberg and Beall, 1990]. Many invertebrates including nematodes, annelids, and molluscs have obliquely striated muscle in which sarcomeres are aligned obliquely to the Z-band-like structures [Rosenbluth, 1965]. The body wall muscle of the nematode *Caenorhabditis elegans* is a representative example and has been extensively studied using genetic and cell biological approaches [Waterston, 1988; Moerman and Fire, 1997; Moerman and Williams, 2006]. The barbed ends of actin filaments are anchored to the dense bodies, which are cone-shaped structures with their bases attached to the plasma membrane [Lecroisey et al., 2007]. The base of the dense body is an integrin-based attachment structure and resembles the costamere in cross-striated muscle. The cytoplasmic portion of the dense body is enriched in α-actinin and anchors actin filaments similarly to the Z-bands in cross-striated muscle. Thus, striated muscles in vertebrates and invertebrates share many common structural and functional features, and studies in these different organisms have contributed to understanding the dynamics and organizations of actin filaments in striated muscle.

## Dynamics of Actin Filaments During Myofibril Assembly

Myofibril assembly during muscle differentiation is a major morphogenetic transformation of the actin cytoskeleton. In skeletal myoblasts, actin is a component of stress fibers that lack clear striated organization, while some components including α-actinin and nonmuscle myosin are localized in a striated pattern [Obinata et al., 1966; Pudney and Singer, 1980]. As the cells fuse to form myotubes, actin filaments are registered to the Z-bands and laterally aligned with uniform length and polarity. As myotubes grow, myofibrils expand by adding sarcomeric actin filaments from the sides and at the tips of growing myotubes [Sanger et al., 2006]. Actin filaments in cardiac muscle are similarly assembled during myofibrillogenesis [Rhee et al., 1994; LoRusso et al., 1997]. Myofibrils in differentiated cardiac myocytes exhibit greater levels of plasticity than those in skeletal muscle. Beating cardiac myocytes still undergo mitosis, and myofibrils disassemble during cell division [Ahuja et al., 2004]. When cardiac myocytes are detached from the substrates, they first disassemble myofibrils and then reassemble myofibrils or newly assemble myofibrils after reattachment on appropriate substrates [Sanger et al., 1984; Imanaka-Yoshida et al., 1996]. During this process, actin is initially organized in a nonstriated manner, originally called stress fiber-like structures [Dlugosz et al., 1984]. However, a number of muscle isoforms of sarcomeric proteins are localized to these structures, and they are defined as premyofibrils and nascent myofibrils [Sanger et al., 2006, 2010], in which actin remains nonstriated while α-actinin shows periodical punctate localization (Figs. 1A and 1B). In the early phase of myofibril assembly, actin filaments are also associated with components of I-bands and Z-bands before they are associated with immature thick filaments [Schultheiss et al., 1990]. These structures are termed I-Z-I bodies, which are believed to be precursors of I-band:Z-band:I-band structures [Holtzer et al., 1997]. In *C. elegans* embryonic muscle, actin and myosin assemble separately into distinct filaments before striated myofibrils are formed [Epstein et al., 1993]. However, the mechanism of initial assembly of thin filaments is currently unknown.

In addition to this striking morphogenetic reorganization of actin filaments, the ratios of actin monomer to polymer change drastically during muscle development [Shimizu and Obinata, 1986] (Fig. 1). In 10-day-old embryonic chick skeletal muscle, α-, β-, and γ-actin are expressed [Shimizu and Obinata, 1980] and 1 mg/mL (24 μM) of actin, which is ∼40% of total actin, is maintained in a monomeric form (G-actin) [Shimizu and Obinata, 1986]. However, in 20-day-old muscle, the total amount of actin is tripled primarily due to predominant expression of skeletal muscle α-actin, whereas G-actin is reduced to 0.4 mg/mL (10 μM), which is 5% of total actin [Shimizu and Obinata, 1986]. In adult muscle, the G-actin concentration is reduced further to 0.05 mg/mL (1 μM), which is less than 1 % of total actin and at a level near the critical concentration of purified actin (0.2–0.3 μM for actin alone and 0.6 μM for barbed-end-capped actin) [Shimizu and Obinata, 1986]. These biochemical transitions of actin indicate that polymerization of actin is negatively regulated to maintain high concentrations of G-actin in embryonic muscle whereas actin rapidly polymerizes in late embryonic muscle as myofibrils expand and become mature.

In embryonic muscle, actin depolymerizing factor (ADF)/cofilin, profilin, and β-thymosin, proteins which increase G-actin concentrations, are abundantly expressed [Abe and Obinata, 1989; Abe et al., 1989; Ohshima et al., 1989; Nagaoka et al., 1996; Obinata et al., 1997]. In particular, the functional significance of ADF/cofilin in striated muscle has been described in several organisms (see below). These G-actin binding proteins can cooperate with other F-actin binding proteins including myosin to modulate actin polymerization in a spatially and temporally regulated manner during muscle development. However, how actin polymerization is regulated precisely to promote ordered assembly of myofibrils is not clearly understood.

## Dynamics of Actin Filaments in Mature Myofibrils

Actin in mature striated myofibrils had been considered as a very stable structural component. The half-life of actin in striated muscle was reported as on the order of days [Zak et al., 1977], while an exchange rate of actin subunits within myofibrils with free G-actin was estimated to be on the order of hours [Martonosi et al., 1960]. This estimate is indeed consistent with a recent observation in cultured quail skeletal myotubes in which actin filaments in mature myofibrils are resistant to hours of treatment with latrunculin A, a drug that induces depolymerization of dynamic actin filaments by sequestering G-actin [Wang et al., 2005b]. However, nascent myofibrils and newly assembled mature myofibrils near the cell periphery are sensitive to latrunculin A [Wang et al., 2005b]. Moreover, direct observations of microinjected fluorescently labeled actin have demonstrated that mature sarcomeric actin filaments in cardiac myocytes are capable of rapidly incorporating exogenously microinjected G-actin within minutes without altering the overall organization of myofibrils [Glacy, 1983; McKenna et al., 1985; Dome et al., 1988; LoRusso et al., 1992; Imanaka-Yoshida et al., 1993; Hayakawa et al., 1996; Shimada et al., 1997; Suzuki et al., 1998; Littlefield et al., 2001]. Similar studies using green fluorescent protein (GFP)-tagged actin agree with these observations [Wang et al., 2005a; Bai et al., 2007; Sanger et al., 2009; Skwarek-Maruszewska et al., 2009] with a particular emphasis that it occurs in vivo in zebrafish [Sanger et al., 2009]. However, sites of myofibril incorporation of free actin are different depending on experimental techniques or cell types, and it requires further mechanistic studies to understand how actin is exchanged in and out of myofibrils [Littlefield and Fowler, 1998]. Nonetheless, multiple studies agree that free actin is initially incorporated at either pointed or barbed ends or both ends of sarcomeric actin filaments and eventually spread over the entire I-band [McKenna et al., 1985; Dome et al., 1988; Shimada et al., 1997; Littlefield et al., 2001]. These observations indicate that ends of sarcomeric actin filaments are capable of polymerizing and depolymerizing even in the presence of capping protein (CapZ) and tropomodulin that cap barbed and pointed ends, respectively (see below). Experiments to disturb selectively either barbed or pointed end of actin filaments indicated that the length of actin filaments is altered when capping of pointed ends but not barbed ends is disturbed [Gregorio et al., 1995; Littlefield et al., 2001; Mardahl-Dumesnil and Fowler, 2001; Bai et al., 2007]. Thus, regulation of actin dynamics at the actin-filament pointed end is suggested as an important mechanism to maintain constant length of sarcomeric actin filaments [Littlefield and Fowler, 2008].

A recent study on dynamics of GFP-tagged actin in cardiac myocytes has identified a novel mode of actin filament turnover in myofibrils. Previous fluorescence recovery after photobleaching (FRAP) studies on fluorescein- or rhodamine-labeled actin showed that fluorescently labeled sarcomeric actin filaments recover their fluorescence after photobleaching [Suzuki et al., 1998; Hasebe-Kishi and Shimada, 2000]. However, the recovery appears to saturate in 2 h at 50–60% of the prebleached level of fluorescence, suggesting the presence of two filament populations: dynamic filaments recovering fluorescence rapidly and stable filaments that do not or only slowly recover fluorescence. A comparative study in quail skeletal myotubes and zebrafish skeletal muscle also agrees with these observations [Sanger et al., 2009]. A recent FRAP study using GFP-actin not only confirmed these results but also demonstrated that fluorescence recovery of the dynamic filament population depends on muscle contractility [Skwarek-Maruszewska et al., 2009]. Rapid fluorescence recovery of GFP-actin occurs in beating cardiac myocytes but not in myocytes that had spontaneously stopped beating or had been treated with inhibitors of contractility. Patterns of fluorescence recovery suggest that entire actin filaments rather than just actin subunits at each end turn over. Latrunculin A specifically eliminates the dynamic filament population within sarcomeres. Although the mechanism and significance of this new mode of contractility dependent sarcomeric actin turnover are not understood, this process may promote maturation of myofibrils, or eliminate any misaligned or damaged filaments from contracting myofibrils. Consistent with these observations, muscle contractility is required for assembly of striated organization of myofibrils [Soeno et al., 1999; De Deyne, 2000; Ramachandran et al., 2003; Kagawa et al., 2006]. However, whether actin dynamics are perturbed by inhibition of contractility during myofibril assembly is unknown.

## Pathological Alterations of Sarcomeric Actin Filaments

Alterations of sarcomeric actin filaments occur under pathological conditions influenced by genetic and/or environmental factors. Congenital myopathies are genetic muscle disorders that are characterized by weak skeletal muscle and the presence of rods or aggregates containing actin and other myofibrillar proteins [Clarkson et al., 2004]. Sarcomres are normally formed under these conditions, but some types of myopathies exhibit “cores” where disorganized sarcomeres are found [Sewry, 2008]. The most common form of nondystrophic congenital myopathies is nemaline myopathy. Nemaline myopathy is characterized by the presence of nemaline rods, which are long (1–7 μm) cytoplasmic rods containing actin and Z-band proteins. When the rods are found in the nuclei, these cases are classified as intranuclear rod myopathy. Whether actin in nemaline rods are derived from sarcomeres or newly synthesized molecules is unknown. Nemaline myopathy is caused by mutations in skeletal muscle α-actin (*ACTA1*) [Nowak et al., 1999; Ilkovski et al., 2001] or other actin-associated proteins including tropomyosin (*TPM2* or *TPM3*) [Laing et al., 1995; Donner et al., 2002], nebulin, (*NEB*) [Pelin et al., 1999], troponin T (*TNNT1*) [Johnston et al., 2000], and cofilin-2 (*CFL2*) [Agrawal et al., 2007]. Actin myopathy is another type of congenital myopathy, which is characterized by the presence of excess actin filament inclusions and typically caused by mutations in *ACTA1*. A number of myopathy-causing mutations have been identified in skeletal muscle α-actin, which are located throughout the molecule [Feng and Marston, 2009; Laing et al., 2009]. These actin mutations cause a wide range of molecular defects in protein folding, binding to actin-binding proteins, polymerization, and cytoskeletal organization, and it has been difficult to correlate the molecular defect with severity of the disease [Costa et al., 2004; Bathe et al., 2007; Vandamme et al., 2009a, b]. A mutant form of actin with defective polymerization or depolymerization could impair normal actin filament turnover in muscle, but whether a defect in actin dynamics is a major cause of congenital myopathies remains to be determined.

Many of the myopathy-causing actin mutants induce actin rods or aggregates when they are exogenously expressed in muscle or nonmuscle cells [Costa et al., 2004; Bathe et al., 2007; Domazetovska et al., 2007; Vandamme et al., 2009a, b, c]. Although these actin rods and aggregates morphologically resemble nemaline rods in patient muscle, the presence of α-actinin, a component of in vivo nemaline rods, is variable in cell culture models. Some actin mutants induce α-actinin-positive actin rods, while other mutants induce α-actinin-negative actin rods [Bathe et al., 2007; Domazetovska et al., 2007; Vandebrouck et al., 2010]. A recent study has shown that α-actinin-negative actin rods have ADF/cofilin and are similar to stress-induced ADF/cofilin-actin rods in the absence of actin mutations [Vandebrouck et al., 2010]. ADF/cofilin-actin rods have been detected in a wide variety of eukaryotic cells and are often induced under stress or pathological conditions [Bamburg and Wiggan, 2002; Ono, 2007]. In cultured vertebrate skeletal myoblasts and myotubes, formation of ADF/cofilin-actin rods is induced by treatment with dimethylsulfoxide within 1 h [Abe et al., 1993; Ono et al., 1993] or by increasing the concentration of ADF/cofilin by overexpression or microinjection [Hosoda et al., 2007; Nagaoka et al., 1995; Ono et al., 1996]. ADF/cofilin-actin rods can also be induced in the *C. elegans* striated muscle by overexpression of ADF/cofilin [Ono and Ono, 2009]. Thus, formation of ADF/cofilin-actin rods appears to be a conserved actin reorganization process in muscle, but its biological significance and relationship to myopathy rods are not clearly understood. Pathological roles of rods in neurodegenerative diseases [Minamide et al., 2000; Whiteman et al., 2009] and a protective role against stress by conserving cellular ATP usage [Bernstein et al., 2006] have been proposed in other cell types. However, whether these roles of rods also apply to muscle is not known. It will also be interesting to determine whether nemaline rods and ADF/cofilin-actin rods are mutually exclusive or functionally related structures.

## Regulators of Actin Filament Dynamics in Striated Muscle

### Enhancers of Actin Filament Turnover

Although actin by itself can spontaneously polymerize and depolymerize in vitro, its turnover rate is too slow to support rapid cytoskeletal reorganization in many dynamic cellular processes in vivo. A number of actin-binding proteins have activities in enhancing actin filament turnover by promoting polymerization, depolymerization, or filament severing [Paavilainen et al., 2004; Nicholson-Dykstra et al., 2005; Ono, 2007]. Among them, ADF/cofilin plays an essential role in organized assembly of sarcomeric actin filaments [Ono, 2003a, 2007]. ADF/cofilin enhances actin filament turnover by severing actin filaments and promoting dissociation of actin monomers from the pointed ends [Van Troys et al., 2008]. Mammals have three ADF/cofilin isoforms: ADF (destrin), cofilin-1 (nonmuscle cofilin), and cofilin-2 (muscle-cofilin) [Matsuzaki et al., 1988; Moriyama et al., 1990; Ono et al., 1994]. Birds have two isoforms: ADF and cofilin [Abe et al., 1990; Adams et al., 1990]. In mammals, cofilin-2 is the predominant isoform in skeletal muscle, while cofilin-1 and cofilin-2 are expressed in cardiac muscle [Ono et al., 1994; Thirion et al., 2001; Vartiainen et al., 2002]. Expression of cofilin-2 is detected early in myogenesis in the mouse myotome and limb buds [Mohri et al., 2000]. In birds (chickens), ADF and cofilin are expressed in embryonic skeletal muscle, but only the expression of cofilin persists in adult muscle [Abe and Obinata, 1989; Abe et al., 1989]. Although ADF and cofilin similarly sever and depolymerize actin filaments and bind to G-actin, cofilin has much weaker actin-monomer sequestering activity than ADF [Vartiainen et al., 2002; Yeoh et al., 2002; Chen et al., 2004]. Mouse cofilin-2 has higher affinity for G- and F-actin than cofilin-1 [Vartiainen et al., 2002; Nakashima et al., 2005]. However, whether these biochemical differences among ADF/cofilin isoforms are related to muscle-specific function is unknown.

Microinjection of high concentrations of cofilin into skeletal myotubes induces disruption of sarcomeres and formation of cytoplasmic actin-cofilin rods [Nagaoka et al., 1995]. However, the activity of microinjected cofilin is gradually suppressed over time. In nonmuscle cells, phosphorylation of ADF/cofilin at conserved Ser-3 inactivates its actin-regulatory activities [Van Troys et al., 2008]. In muscle cells, an S3A mutant of cofilin that cannot be phosphorylated is still inactivated over time, and direct binding of phosphoinositides to cofilin has been proposed as a regulatory mechanism [Hosoda et al., 2007]. Recently, muscle LIM protein has been shown to bind to cofilin-2 and enhance the cofilin-2 activity, which might be a novel mechanism to regulate actin dynamics [Papalouka et al., 2009]. Depletion of cofilin from cardiac myocytes by RNA interference causes disorganization of sarcomeric actin filaments [Skwarek-Maruszewska et al., 2009]. Thus, these observations suggest that cofilin is important for maintenance of sarcomeric actin organization and that its activity is under control.

Isoform-specific and muscle-specific function of ADF/cofilin in actin filament organization has also been demonstrated in the nematode *C. elegans*. *C. elegans* has two ADF/cofilin isoforms, UNC-60A and UNC-60B, which are generated by the *unc-60* gene by alternative splicing [McKim et al., 1994; Anyanful et al., 2004]. UNC-60A and UNC-60B are ∼30% identical in their amino acid sequences and show no specific correlation to mammalian ADF/cofilin isoforms based on sequence comparison. UNC-60A has very weak actin-filament severing activity but has strong actin-monomer sequestering activity. In contrast, UNC-60B has strong actin-filament severing activity but has very weak actin-monomer sequestering activity [Ono and Benian, 1998; Yamashiro et al., 2005; Ono et al., 2008]. UNC-60B is specifically expressed in striated muscle, and loss-of-function and null mutations of *unc-60B* cause severe disorganization of sarcomeric actin filaments from embryonic stages [Ono et al., 1999, 2003]. Actin filament severing activity of UNC-60B can be eliminated without altering its G-actin binding activity by mutations near the C-terminus [Ono et al., 2001], and these mutants fail to function properly in organizing sarcomeric actin [Ono et al., 1999, 2008], indicating that actin filament severing by UNC-60B is important for sarcomeric actin organization. Furthermore, actin-interacting protein 1 (AIP1) that is encoded by the *unc-78* gene cooperates with UNC-60B to disassemble actin filaments and is essential for sarcomeric actin organization [Ono, 2001; Mohri and Ono, 2003; Mohri et al., 2004, 2006; Ono et al., 2004]. Although AIP1 is an evolutionarily conserved partner of ADF/cofilin in regulation of actin dynamics [Ono, 2003b], its function in vertebrate muscle has not been reported.

Functional significance of cofilin in muscle has recently been further emphasized by the finding that a mutation in the human cofilin-2 gene (*CFL2*) causes nemaline myopathy [Agrawal et al., 2007]. The mutation converts Ala-35 into Thr and significantly reduces the cofilin-2 protein level in skeletal muscle and impairs solubility when recombinant cofilin-2 is expressed in a bacterial system, suggesting that the myopathy-causing mutation is a loss-of-function mutation. In the patient muscle, actin is abnormally accumulated in two kinds of distinct structures: nemaline bodies that contain components of the Z-bands and separate aggregates that do not contain α-actinin [Agrawal et al., 2007]. To date, mutation in *CFL2* is rare and found only in two patients who are siblings in a family of Middle Eastern origin [Agrawal et al., 2007]. Now that *CFL2* is known as a disease gene, mutations in *CFL2* can be investigated in a targeted manner. Additional clinical studies on cofilin-dependent nemaline myopathy and development of a mouse model of this disease should provide insight into the pathogenesis of nemaline myopathy as well as the normal function of cofilin in sarcomeric actin assembly and maintenance.

Gelsolin is a member of another class of actin-severing proteins that sever actin filaments and cap their barbed ends in a calcium-dependent manner [Sun et al., 1999; McGough et al., 2003; Silacci et al., 2004]. Although expression of gelsolin in striated muscle has been reported for many years, its role in regulation of sarcomeic actin is not clearly understood. Despite the fact that gelsolin strongly severs and caps purified muscle actin filaments in vitro, it localizes along the thin filaments in vertebrate striated muscle [Yin et al., 1981; Rouayrenc et al., 1984; Carron et al., 1986; Dissmann and Hinssen, 1994]. Purified gelsolin can bind to the side of thin filaments without severing them in permeabilized skeletal muscle cells [Gonsior and Hinssen, 1995]. In vitro, tropomyosin protects actin filaments from severing by gelsolin [Fattoum et al., 1983; Ishikawa et al., 1989] and directly binds to gelsolin [Maciver et al., 2000]. However, in permeabilized muscle cells, gelsolin can still bind to the side of thin filaments without severing even after extraction of tropomyosin by a high-salt buffer [Gonsior and Hinssen, 1995], and the regulatory mechanism of gelsolin in muscle is currently unknown.

Besides gelsolin, a number of gelsolin-related proteins are present, and some of them are expressed in striated muscle [Ono, 2007]. Conventional gelsolin has six homologous domains of 100–120 amino acids [Kwiatkowski et al., 1986]. Flightless-1 is a conserved gelsolin-related protein with six gelsolin-like repeats and additional leucine-rich repeats at the N-terminus [Campbell et al., 1993]. Flightless-1 mutants in *Drosophila* have disorganized myofibrils in the indirect flight muscle [Miklos and De Couet, 1990]. Similarly, mutations of a Flightless-1 homolog (FLI-1) in *C. elegans* causes actin filament disorganization in striated muscle [Deng et al., 2007; Lu et al., 2008]. One report shows that the gelsolin-related portion of *C. elegans* FLI-1 severs actin filaments in a calcium-independent manner [Goshima et al., 1999]. However, the biochemical activity of Flightless-1 has not been extensively characterized, and its actin-regulatory function is not clearly understood. In addition, *C. elegans* has gelsolin-like protein-1 (GSNL-1) with four gelsolin-like repeats and calcium-dependent actin filament severing and capping activities [Klaavuniemi et al., 2008; Liu et al., 2010]. Although GSNL-1 is enriched in striated muscle [Fox et al., 2007], a gene knockout of GSNL-1 does not cause a detectable phenotype in sarcomeric actin organization [T. Klaavumiemi and S. Ono, unpublished data], and its function in muscle is currently unknown.

### G-Actin-Binding Proteins

Proteins that bind to G-actin influence actin dynamics by altering polymerization/depolymerization kinetics and exchange rates of actin-bound ATP or ADP [Paavilainen et al., 2004]. Vertebrate striated muscle expresses major G-actin-binding proteins such as thymosin β4 and profilin, but levels of these proteins decrease during muscle development [Babcock and Rubenstein, 1993; Nagaoka et al., 1996]. In adult skeletal muscle, G-actin concentrations are near the level of critical concentration of purified actin [Shimizu and Obinata, 1986], and functions of these G-actin-binding proteins are not clearly understood. Nonetheless, vertebrates have isoforms of profilin (profilin II) [Honore et al., 1993] and cyclase-associated protein (CAP2) [Bertling et al., 2004; Peche et al., 2007; Wolanski et al., 2009] that are highly expressed in striated muscle, suggesting that they have specific functions in muscle. *C. elegans* has three profilin isoforms, and two of them (PFN-2 and PFN-3) are expressed in striated muscle [Polet et al., 2006]. Although profilin-null mutations cause only minor alterations in sarcomeric actin organization, it enhances actin disorganization when tropomodulin, a pointed-end capping protein, is also depleted [Yamashiro et al., 2008]. Profilin inhibits actin elongation at the pointed ends [Pantaloni and Carlier, 1993]. Therefore, profilin and tropomodulin may cooperatively regulate actin dynamics at the pointed ends. Two cyclase-associated protein isoforms are present in *C. elegans*, and one of them (CAS-1) is expressed in striated muscle [S. Ono, unpublished data]. However, the function of CAS-1 is currently unknown.

### F-Actin-Side-Binding Proteins

Proteins that bind to the side of actin filaments are integral components of sarcomeric thin filaments and commonly stabilize actin filaments. Tropomyosin is perhaps the best characterized side-binding protein that regulates muscle contraction as well as actin filament stability [Gunning et al., 2008]. Tropomyosin by itself stabilizes actin filaments in vitro by inhibiting spontaneous actin polymerization and depolymerization [Lal and Korn, 1986; Hitchcock-DeGregori et al., 1988; Broschat et al., 1989; Broschat, 1990]. Furthermore, tropomyosin protects actin filaments from severing by ADF/cofilin [Bernstein and Bamburg, 1982; Ono and Ono, 2002] or gelsolin [Fattoum et al., 1983; Ishikawa et al., 1989]. In *C. elegans* striated muscle, tropomyosin functions antagonistically to ADF/cofilin and AIP1 to stabilize sarcomeric actin organization [Ono and Ono, 2002; Yu and Ono, 2006]. Importantly, a number of mutations in three of the four tropomyosin genes are associated with human heart and skeletal muscle diseases [Kee and Hardeman, 2008; Wieczorek et al., 2008]. Mutations in *TPM1* cause hypertrophic and dilated cardiomyopathies [Thierfelder et al., 1994; Watkins et al., 1995; Olson et al., 2001]. Mutations in *TPM2* cause nemaline myopathy [Donner et al., 2002], distal arthrogryposis (type 1: DA1) [Sung et al., 2003], and cap myopathy [Lehtokari et al., 2007]. Mutations in *TPM3* cause nemaline myopathy [Laing et al., 1995] and cap myopathy [De Paula et al., 2009]. Most of the disease-causing mutations in tropomyosin are genetically dominant, in that heterozygous carriers have disease. Biochemical and physiological studies on mutant tropomyosins have revealed impaired muscle contraction and relaxation, but the effects of the mutations on actin dynamics have not been investigated. A missense mutation (M9R) in *TPM3* causes nemaline myopathy and reduces affinity of tropomyosin with tropomodulin, a pointed-end capping protein, thereby potentially influencing the actin dynamics at the pointed ends [Akkari et al., 2002; Ilkovski et al., 2008; Gokhin et al., 2010] (see below for functions of Tmod).

Nebulin is a large protein (600–800 kDa) that has been proposed to be a ruler that determines lengths of sarcomeric thin filaments [McElhinny et al., 2003]. Nebulin is expressed at high levels in skeletal muscle, while it is at low levels in cardiac muscle [Kazmierski et al., 2003]. A nebulin-like protein, nebulette (107 kDa), is expressed in cardiac muscle and may have an overlapping function with nebulin [Moncman and Wang, 1995]. Nebulin is associated with sarcomeric thin filaments, and a single nebulin molecule spans the entire length of the filament [Wright et al., 1993]. Mutations in the human nebulin gene (*NEB*) cause nemaline myopathy [Pelin et al., 1999; Ilkovski, 2008]. The amino acid sequence of nebulin contains ∼200 homologous modules of ∼35 residues, called nebulin-like repeats. Some nebulin fragments containing multiple modules exhibit stoichiometric binding to actin that corresponds to one module per actin subunit [Zhang et al., 1998]. These fragments can promote actin polymerization under low-salt conditions in which actin alone does not polymerize [Chen et al., 1993; Gonsior et al., 1998; Root and Wang, 2001], and also inhibit actin depolymerization [Chen et al., 1993]. Antibody-inhibition or depletion of nebulin disturbs organized assembly of sarcomeric actin filaments in cultured skeletal myotubes [Nwe and Shimada, 2000; McElhinny et al., 2005]. Furthermore, depletion of nebulin from cardiac myocytes induces elongation of actin filaments from their pointed ends [McElhinny et al., 2005]. These observations support the “nebulin ruler” hypothesis for regulation of thin-filament lengths. However, in cardiac muscle, the expression level of nebulin is so low that a single nebulin molecule would need to regulate multiple thin filaments [Horowits, 2006]. Castillo et al. recently reported that nebulin does not extend to the pointed ends of thin filaments in rabbit skeletal muscle [Castillo et al., 2009]. Furthermore, in nebulin-deficient mice, thin filaments are assembled with uniform but shorter length than those in wild-type in neonatal skeletal muscle, becoming more variable in length during postnatal development [Bang et al., 2006; Witt et al., 2006]. Similarly, short thin filaments are found in human nemaline myopathy patients with nebulin mutations [Ottenheijm et al., 2009]. These observations reinforce the idea that nebulin stabilizes thin filament lengths by preventing actin disassembly. However, since thin filament lengths are still regulated, nebulin alone does not determine length of thin filaments. Nebulin also binds to capping protein (CapZ) [Pappas et al., 2008] and tropomodulin [McElhinny et al., 2001], which are actin barbed-end and pointed-end capping proteins, respectively. A new study has demonstrated that nebulin stabilizes sarcomeric thin filaments not only by inhibiting actin depolymerization but also by stabilizing other thin filament proteins including tropomyosin and tropomodulin [Pappas et al., 2010]. Thus, these recent functional studies on nebulin strongly suggest that nebulin is not a strict ruler for thin filaments but rather a regulator of dynamic exchange of actin subunits and other thin filament proteins.

Several actin-binding proteins with multiple immunoglobulin-like (Ig) repeats are expressed in striated muscle and associated with myofibrillar actin filaments. The palladin/myopalladin/myotilin family of Ig-repeat proteins localize to the Z-band and play important roles in sarcomeric organization [Otey et al., 2005, 2009]. Myotilin is highly expressed in skeletal muscle [Salmikangas et al., 1999], while myopalladin is enriched in cardiac muscle [Bang et al., 2001]. Palladin is widely expressed in both muscle and nonmuscle tissues in embryos, but its expression is diminished in adult muscle [Parast and Otey, 2000]. Myotilin and palladin directly bind to actin filaments, and myotilin stabilizes actin filaments by inhibiting spontaneous depolymerization in vitro [Salmikangas et al., 2003; Dixon et al., 2008]. Mutations in the human myotilin gene (*MYOT*) cause myotilinopathy that includes three known types of skeletal muscle diseases: limb girdle muscular dystrophy type 1A, myofibrillar myopathy, and spheroid body myopathy. Myotilinopathy patients commonly exhibit progressive disorganization of sarcomeres with disarrayed Z-bands [Olive et al., 2005], suggesting that myotilin is important for stability of sarcomeres at the Z-bands. However, disease-causing mutations in myotilin do not affect actin-binding activity of myotilin [von Nandelstadh et al., 2005]. Myotilin binds to other actin-binding proteins including α-actinin and filamin and may function as an adaptor molecule at the Z-bands. A mutation in myopalladin is associated with dilated cardiomyopathy [Duboscq-Bidot et al., 2008], but how this mutation affects myotilin interactions with actin and its role in actin filament dynamics are not understood. Thus, the palladin/myopalladin/myotilin family of proteins clearly has important functions in muscle, but whether they function as stabilizers of actin filaments or adaptors for signaling and structural proteins is a subject of further investigation.

Kettin is a large protein of 500–700 kDa with 30-35 Ig-repeats that is specifically present in invertebrate striated muscle. Kettin directly binds to actin filaments and localizes to the I-band and Z-band [Lakey et al., 1993; Maki et al., 1995; van Straaten et al., 1999; Ono et al., 2006]. Although a direct effect of kettin on actin dynamics in vitro has not been determined, kettin is implicated in assembly and stability of myofibrils in *Drosophila* and *C. elegans*. Kettin is associated with actin filaments during early myofibrillogenesis in *Drosophila* muscle [Ayme-Southgate et al., 2004], and a mutational analysis originally suggested that kettin was essential for myofibril assembly [Hakeda et al., 2000]. However, subsequent genetic studies on the kettin gene have shown that kettin is expressed from the *sallimus* (*sls*) gene that also encodes zormin and connectin/titin by complex alternative splicing [Machado and Andrew, 2000; Zhang et al., 2000; Burkart et al., 2007]. Connectin/titin is particularly known as an important elastic component of sarcomeres [Maruyama, 1997], and interpretation of the *sls* mutant phenotypes is complicated due to defects in multiple sarcomeric proteins. By contrast, *C. elegans* kettin is encoded by *ketn-1* that is a separate gene from other connectin/titin-related genes in *C. elegans* [Ono et al., 2005, 2006]. Depletion of *C. elegans* kettin by RNAi causes disorganization of sarcomeric actin filaments when muscle is subjected to chemically induced hypercontraction, suggesting that kettin is important for stability of sarcomeric actin during contraction. *C. elegans* kettin cooperates functionally with α-actinin and ALP-enigma [Ono et al., 2006; Han and Beckerle, 2009], suggesting that these proteins reinforce stability of actin filaments at the dense bodies.

Xin is a muscle-specific protein in vertebrates that stabilizes actin filaments. Xin contains 15–28 repeats of 16-amino-acid sequence, called Xin repeats. Xin repeats directly bind to actin filaments in vitro and stabilize actin filaments in stress fibers when Xin repeats are expressed in cultured smooth muscle cells [Pacholsky et al., 2004]. Structural analysis demonstrated that Xin repeats bind to the side of actin filaments in a similar manner to nebulin repeats [Cherepanova et al., 2006]. Xin is enriched in intercalated discs in cardiac muscle and myotendenous junctions in skeletal muscle, where actin filaments are anchored to the plasma membrane [Sinn et al., 2002]. Xin also interacts with the cadherin-catenin complex [Choi et al., 2007], and this interaction may promote specific functions of Xin at the actin-membrane interface and functional differentiation between Xin and nebulin. In agreement with these observations, Xin-deficient mice exhibit cardiac myopathy with disorganized intercalated discs [Gustafson-Wagner et al., 2007; Otten et al., 2010; Wang et al., 2010]. Whether Xin functions as an actin-filament stabilizer or a reinforcement of the link between actin and the cadherin-catenin complex has not been determined.

UNC-87 is a calponin-like protein that stabilizes actin filaments and is found only in nematode muscle [Goetinck and Waterston, 1994a]. Mutations of UNC-87 in *C. elegans* cause disorganization of sarcomeric actin filaments in body wall muscle, but this phenotype is suppressed when muscle contraction is reduced, suggesting that UNC-87 stabilizes actin filaments during actomyosin contraction [Goetinck and Waterston, 1994b]. UNC-87 contains seven calponin-like (CLIK) repeats that are found in the C-terminus of mammalian calponin, but it has no calponin-homology (CH) domain. UNC-87 binds directly to the side of actin filaments and stabilizes actin filaments when it is exogenously expressed in mammalian cells [Gimona et al., 2003; Kranewitter et al., 2001]. Furthermore, UNC-87 protects actin filaments from severing by ADF/cofilin in vitro and antagonizes ADF/cofilin in vivo [Yamashiro et al., 2007]. Since *C. elegans* does not have nebulin or Xin, UNC-87 might be a functional homologue of these actin stabilizing proteins in striated muscle.

### Capping Proteins

Both pointed and barbed ends of sarcomeric actin filaments are capped by capping proteins. However, as described above, sarcomeric actin filaments are capable of exchanging actin monomers at both ends, indicating that the filaments have dynamic caps rather than permanent caps. The barbed ends of sarcomeric actin filaments are capped by CapZ (also known as capping protein or β-actinin) and aligned in register at the Z-band [Casella et al., 1987]. During myofibril assembly, CapZ is absent from premyofibrils and becomes associated with nascent myofibrils in a nonstriated manner and gradually assembled into striated Z-bands in nascent myofibrils prior to the striated appearance of actin filaments [Wang et al., 2005; Schafer et al., 1993]. Inhibition of CapZ by microinjection of an antibody or by expression of a dominant-negative CapZ mutant disturbs assembly of sarcomeric actin filaments into myofibrils in cultured skeletal myotubes [Schafer et al., 1995]. Transgenic expression in mouse heart of a CapZ mutant that binds poorly to actin causes myofibril disorganization and cardiac defects [Hart and Cooper, 1999]. These observations suggest that CapZ is important for regular alignment of the actin barbed ends at the Z-bands. However, the mechanism of this function is still unclear. CapZ binds directly to the C-terminus of nebulin, and this interaction is one mechanism for proper alignment of actin barbed ends and CapZ at the Z-bands [Witt et al., 2006; Pappas et al., 2008]. CapZ also binds to α-actinin [Papa et al., 1999], although the functional significance of this interaction is not understood. Limiting actin elongation at the barbed end is probably another important function of CapZ. CapZ itself is dynamic in the Z-band of cardiomyocytes [Hartman et al., 2009]. Thus, dissociation of CapZ from actin can allow incorporation of actin monomers at the barbed end. Therefore, actin elongation and CapZ dynamics must be balanced to maintain alignment of actin-filament barbed ends at the Z-band. In cardiomyocytes, hypertrophic stimuli, endothelin-1 and phenylephrine, enhance CapZ dynamics, and phosphatidylinositol 4,5-bisphosphate (PIP2) and protein kinase C mediate this enhancement [Hartman et al., 2009]. PIP2 directly binds to CapZ and inhibits its capping activity [Heiss and Cooper, 1991], but whether PIP2 is able to dissociate CapZ from the actin barbed end in vitro is controversial [Kim et al., 2007; Kuhn and Pollard, 2007]. A number of proteins are known to regulate capping protein in nonmuscle cells, but their functions in striated muscle are largely unknown [Cooper and Sept, 2008]. Therefore, PIP2 and other CapZ regulators may collaborate to regulate the dynamics of CapZ in striated muscle.

The pointed ends of sarcomeric actin filaments are capped by tropomodulin (Tmod) [Fowler et al., 1993]. Tmod caps the actin pointed ends and inhibits both polymerization and depolymerization. During myofibril assembly in cultured embryonic chick skeletal myotubes and embryonic heart, Tmod is associated with actin filaments before striation of actin is established [Almenar-Queralt et al., 1999]. A similar localization pattern is observed in the mouse embryonic heart [Fritz-Six et al., 2003; Ehler et al., 2004]. However, during reassembly of myofibrils in cultured embryonic chick cardiac myocytes, Tmod is assembled into myofibrils after actin and tropomyosin have already established a striated organization [Gregorio and Fowler, 1995], suggesting that the cardiac-myocyte model is somewhat different from in vivo muscle in the regulation of Tmod localization. Genetic studies show that Tmod is important for myofibril assembly in the mouse heart [Chu et al., 2003; Fritz-Six et al., 2003; McKeown et al., 2008], *Drosophila* [Bai et al., 2007], and *C. elegans* [Stevenson et al., 2007; Yamashiro et al., 2008]. A critical role of Tmod in regulating the length of thin filaments has been demonstrated in multiple experimental systems. Overexpression of Tmod shortens thin filaments, whereas knockdown or inhibition of capping activity of Tmod elongates thin filaments [Gregorio et al., 1995; Sussman et al., 1998; Littlefield et al., 2001; Mardahl-Dumesnil and Fowler, 2001; Bai et al., 2007]. These results suggest that Tmod regulates actin elongation at the pointed ends in sarcomeres. Tmod is dynamic at the pointed ends under normal conditions, while Tmod overexpression stabilizes the pointed-end capping and prevents elongation. In contrast, loss of the Tmod cap allows excessive elongation of actin at the pointed ends. Intriguingly, *Tmod1* null cardiac myocytes develop fewer and thinner myofibrils than wild-type cells but with sarcomeric organization of uniformly aligned actin filaments [Ono et al., 2005], suggesting that a Tmod-independent mechanism to regulate the length of thin filaments exists as proposed previously [Gregorio and Fowler, 1995; Gregorio and Fowler, 1996].

Tmod cooperates with other actin-regulatory proteins and regulates length or stability of thin filaments. Tropomyosin binds to Tmod and enhances its capping activity [Fischer and Fowler, 2003]. A missense mutation (M9R) in *TPM3* causes nemaline myopathy and reduces the affinity of tropomyosin for Tmod [Akkari et al., 2002; Greenfield and Fowler, 2002; Ilkovski et al., 2008]. Interestingly, inhibition of the Tmod-tropomyosin interaction by antibody injection induces disassembly of thin filaments [Mudry et al., 2003]. It is accompanied by dissociation of tropomyosin, suggesting that Tmod stabilizes tropomyosin at the pointed end and protects actin filaments from depolymerization and severing. However, this effect of Tmod-tropomyosin inhibition is remarkably different from that of simple knockdown of Tmod, and whether this effect is independent of its capping activity still needs to be determined. Pointed-end capping by Tmod inhibits actin depolymerization induced by ADF/cofilin in vitro [Yamashiro et al., 2008]. However, in *C. elegans* muscle, TMD-1 (*C. elegans* Tmod) appears to cooperate with ADF/cofilin and AIP1 in sarcomeric organization of actin filaments [Yamashiro et al., 2008]. Both Tmod and ADF/cofilin might function to maintain constant length of actin filaments by preventing excessive actin elongation at the pointed-end. In contrast, *Drosophila* sarcomere length short (SALS) functions to enhance elongation of thin filaments by antagonizing the activity of Tmod [Bai et al., 2007]. In the *Drosophila* striated muscle, knockdown of SALS shortens the length of thin filaments, while overexpression of SALS elongates thin filaments from their pointed ends. The *sals* gene encodes two SALS isoforms that have one or two WASP homology 2 (WH2) domains. However, in vitro, a SALS fragment containing two WH2 domains binds to the actin pointed ends and inhibits elongation rather than promoting elongation as expected from the in vivo observations. Therefore, the precise mechanism of SALS-mediated thin filament elongation remains unclear. An ortholog of SALS appears to be absent in vertebrates and nematodes, and it remains to be determined whether a functional homolog of SALS exists in other organisms.

Leiomodin (Lmod) is a Tmod-related protein with a unique C-terminal extension containing a poly-proline sequence and a WH2 domain [Conley et al., 2001]. Vertebrates have three Lmod isoforms (Lmod1, Lmod2, and Lmod3). Lmod1 is expressed in smooth muscle, while Lmod2 is specifically expressed in striated muscle [Conley et al., 2001; Tsukada et al., 2010]. Lmod3 is a fetal isoform with no known function and only reported in sequence databases. Lmod2 has potent actin nucleation activity [Chereau et al., 2008]. In cardiac myocytes, Lmod2 localizes near the M-line where the actin pointed ends are concentrated. Knockdown of Lmod2 induces disorganization of sarcomeric structures in cultured rat cardiomyocytes [Chereau et al., 2008]. One of major questions in myofibril assembly is how unbranched, long actin filaments are nucleated in muscle cells. Lmod2 nucleates unbranched actin filaments, and it is a candidate for an actin-nucleator in muscle cells. However, expression of Lmod2 is detected in muscle later than expression of Tmod1 [Tsukada et al., 2010], and involvement of Lmod2 in early stages of de novo myofibril assembly still needs to be determined. Rather, a more recent study has shown that Lmod2 binds to the pointed ends of actin filaments without capping [Tsukada et al., 2010]. Importantly, Lmod2 antagonizes Tmod1 and supports actin elongation from the pointed ends against capping by Tmod1 [Tsukada et al., 2010]. Thus, Lmod2 regulates thin filament length in sarcomeres in a similar manner to *Drosophila* SALS.

### Actin Nucleators

As mentioned above, an actin nucleator for initial assembly of thin filaments in muscle cells has not been identified. The Arp2/3 complex nucleates branched actin networks [Goley and Welch, 2006], but it is involved in myoblast fusion and is not implicated in sarcomeric actin filament formation [Richardson et al., 2008; Rochlin et al., 2010]. Since formins are well-characterized promoters of nucleation and elongation of unbranched actin filaments [Paul and Pollard, 2009; Chesarone et al., 2010], they are attractive candidate regulators for initial formation of sarcomeric thin filaments. To date, no formin has been implicated in nucleation and elongation of actin filaments in muscle. Proteins of the formin family share formin-homology 2 (FH2) domains that are responsible for actin-binding. Based on the sequence of the FH2 domains, formins can be classified into seven subfamilies [Higgs, 2005; Higgs and Peterson, 2005]. Among them, the FHOD (also known as FHOS) subfamily of formins is expressed in striated muscle. Mammalian FHOD1 is highly expressed in skeletal muscle [Tojo et al., 2003] and localizes to the Z-bands [S. Blystone, personal communication], but its biochemical activity and cellular function are not known. Mammalian FHOD3 (also known as Fhos2) is highly expressed in the heart [Kanaya et al., 2005], and knockdown of FHOD3 inhibits sarcomere assembly in rat cardiomyocytes in culture [Taniguchi et al., 2009]. However, the mechanism by which FHOD3 regulates myofibril organization remains unknown. Under the conditions tested by Taniguchi et al. [2009], a C-terminal fragment of FHOD3 including the FH2 domain does not nucleate actin filaments in vitro, and FHOD3 is colocalized with sarcomeric actin and unexpectedly concentrated near the actin-filament pointed ends. In *C. elegans*, FHOD-1 is the only formin of the FHOD subfamily, and it is predominantly expressed in several types of muscle cells. FHOD-1-null mutant worms exhibit some thinner striated muscle cells than wild-type worms but show no major abnormalities in sarcomeric actin organization [Pruyne et al., 2009]. Thus, these observations suggest that the FHOD subfamily of formins have an important function in muscle, although its specific function remains elusive. Perhaps, additional information on the biochemical activity of FHOD regulation of actin dynamics, interactions with regulators and/or cofactors, and post-translational modifications may provide clues to understanding the role of FHOD in striated muscle.

### Myosin

Myosin II is the force-generating enzyme in sarcomeres. It is also an important regulator of myofibril assembly. Pharmacological perturbation of myosin motor activity disrupts organized assembly of sarcomeric actin filaments [Soeno et al., 1999; De Deyne, 2000; Ramachandran et al., 2003; Kagawa et al., 2006]. In chicken cardiac myocytes, nonmuscle myosin II is incorporated into nascent myofibrils, but is replaced with muscle-specific myosin II during myofibril maturation [Rhee et al., 1994] (Fig. 1). In mammals, a number of muscle-specific myosin isoforms exist, and embryonic and neonatal isoforms are replaced by adult isoforms during development [Whalen et al., 1981]. In *Drosophila* embryonic muscle, nonmuscle myosin II (*zipper*) is required for formation of striated sarcomeres [Bloor and Kiehart, 2001], suggesting that nonmuscle myosin has a specific function in myofibril assembly. However, the mechanism by which myosin regulates myofibril assembly is not completely understood. The myosin head detects polarity of actin filaments. Therefore, one likely mechanism is that myosin interacts with actin filaments and aligns them with uniform polarity within sarcomeres. Myosin also competes with ADF/cofilin for actin binding [Nishida et al., 1984] and enhances actin polymerization from ADF/cofilin-bound actin monomers in vitro [Abe and Obinata, 1989], suggesting that myosin can initiate actin assembly during myofibrillogenesis. On the other hand, myosin activity enhances actin turnover in mature myofibrils in rat cardiomyocytes [Skwarek-Maruszewska et al., 2009]. In nonmuscle cells, myosin-dependent disassembly or turnover of actin bundles or network has been observed in cytokinesis [Guha et al., 2005; Murthy and Wadsworth, 2005] and cell migration [Medeiros et al., 2006; Wilson et al., 2010]. Moreover, skeletal muscle myosin II can enhance disassembly of actin bundles in vitro by unbundling and subsequent filament depolymerization [Haviv et al., 2008]. However, myosin-dependent actin turnover appears to be limited to a subset of sarcomeric actin filaments [Skwarek-Maruszewska et al., 2009], suggesting that the majority of thin filaments are protected from myosin-dependent disassembly by unknown mechanisms. Further investigations are required to understand the function and regulation of myosin in actin turnover during myofibril assembly and maintenance.

### Perspectives

**Fig. 2:**
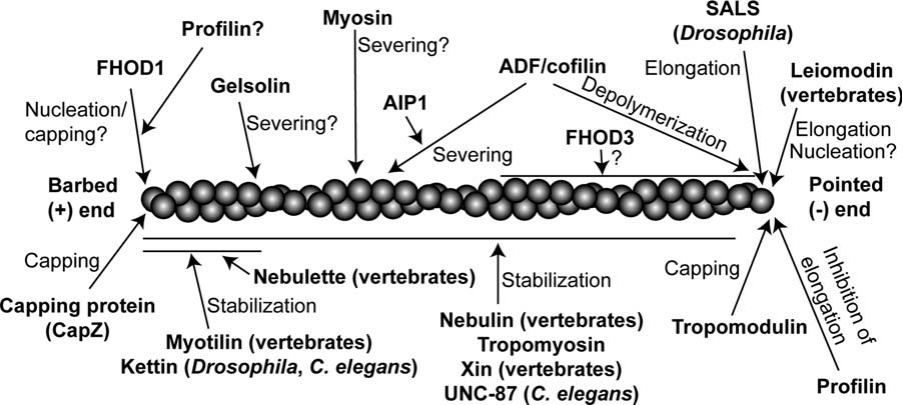
Regulators of actin filament dynamics in striated muscle An actin filament is drawn with its barbed end to the left and pointed end to the right. Regulators shown above the filament can enhance actin dynamics by promoting polymerization, depolymerization, or severing. Regulators shown below the filament stabilize actin filaments.

Studies in live muscle cells have demonstrated that actin in sarcomeres is dynamic during assembly and even in mature myofibrils. A number of regulators of sarcomeric actin dynamics have been identified, and functional studies have revealed their important roles in myofibril assembly, sarcomere organization, and maintenance of myofibrils (Fig. 2). Regulators of sarcomeric actin dynamics can be classified into two types: enhancers of actin dynamics and stabilizers of actin filaments (Fig. 2). Actin-dynamics enhancers (e.g., ADF/cofilin) and actin-filament stabilizers (e.g., tropomyosin) often antagonistically regulate actin turnover. Therefore, a simple model would be that these two types of actin regulators maintain a balance for proper assembly and maintenance of sarcomeric actin organizations. However, the actual process in muscle cells is expected to be much more complex, involving multiple mechanisms at different parts of sarcomeres under different cellular conditions. Importantly, we still do not understand why actin filaments even in mature sarcomeres must be dynamic. Further investigations on the functions of actin-dynamics regulators and physiological significance of actin dynamics in muscle should provide insights into the pathogenesis of muscle diseases as well as the normal mechanism of myofibril assembly and maintenance.
